# FAM76B regulates PI3K/Akt/NF-κB-mediated M1 macrophage polarization by influencing the stability of PIK3CD mRNA

**DOI:** 10.1007/s00018-024-05133-2

**Published:** 2024-02-29

**Authors:** Juan Wang, Xinyue Zhao, Qizhi Wang, Xiaojing Zheng, Dilihumaer Simayi, Junli Zhao, Peiyan Yang, Qinwen Mao, Haibin Xia

**Affiliations:** 1 Laboratory of Gene Therapy, Department of Biochemistry, College of Life Sciences, Shaanxi Normal University, 199 South Chang’an Road, Xi’an, 710062 Shaanxi Province People’s Republic of China; 2 Department of Pathology, School of Basic Medical Science, Ningxia Medical University, Yinchuan, 750004 People’s Republic of China; 3 Department of Pathology, University of Utah, Huntsman Cancer Institute, 2000 Circle of Hope Drive, Salt Lake City, UT 84112 USA

**Keywords:** FAM76B, Macrophage polarization, PI3K, Akt, Inflammatory bowel disease

## Abstract

Macrophage polarization is closely related to inflammation development, yet how macrophages are polarized remains unclear. In our study, the number of M1 macrophages was markedly increased in *Fam76b* knockout U937 cells *vs.* wild-type U937 cells, and FAM76B expression was decreased in M1 macrophages induced from different sources of macrophages. Moreover, *Fam76b* knockout enhanced the mRNA and protein levels of M1 macrophage-associated marker genes. These results suggest that FAM76B inhibits M1 macrophage polarization. We then further explored the mechanism by which FAM76B regulates macrophage polarization. We found that FAM76B can regulate PI3K/Akt/NF-κB pathway-mediated M1 macrophage polarization by stabilizing PIK3CD mRNA. Finally, FAM76B was proven to protect against inflammatory bowel disease (IBD) by inhibiting M1 macrophage polarization through the PI3K/Akt/NF-κB pathway in vivo. In summary, FAM76B regulates M1 macrophage polarization through the PI3K/Akt/NF-κB pathway in vitro and in vivo, which may inform the development of future therapeutic strategies for IBD and other inflammatory diseases.

**Supplementary Information:**

The online version contains supplementary material available at 10.1007/s00018-024-05133-2.

## Introduction

Macrophage polarization plays a very important role in the occurrence, development, and resolution of inflammation, which is a hot research topic in the scientific community. Macrophages are an important, highly plastic part of the innate immune system and can be induced to differentiate into two main phenotypes, M1 (classic) or M2 (alternatively activated), in the face of different pathogens or stimulation [1, 2]. M1 macrophages can be induced by lipopolysaccharide (LPS) or type 1T-helper cytokines (Th1) (such as interferon [IFN]γ) and upregulate the expression of NOS2 [3] and specific cell surface receptors, including MHCII, CD86, and CD80. Moreover, M1 macrophages also produce cytokines, such as interleukin (IL)-6, IL-1β, TNFα, IL-23A, CCL3, and CCL7 [4–6]. M2 macrophages are induced by type 2T-helper cytokines (Th2) (such as IL-4 or IL-13) and lead to the upregulated expression of Arg-1, Fizz1, and Ym-1, as well as the production of cytokines, including IL-10 [1].

The activation of macrophages is strictly regulated and mediated by signaling cascades downstream of toll-like receptors (TLRs) and cytokine receptors [7]. The PI3K/Akt, NF-κB, JNK/STAT, Notch, and TGFβ signaling pathways are all involved in polarizing macrophages and regulate the conversion between the M1 and M2 phenotypes [8, 9]. It has been reported that initiation of the PI3K/Akt signaling cascade is critical for pro- and anti-inflammatory responses [10], and, in macrophages, this pathway is regarded to be a negative modulator of TLRs and NF-κB pathway [11, 12].

Non-specific chemical inhibition of PI3K in TLR-activated cells can enhance NF-κB activation and NOS2 expression, thereby promoting M1 macrophage polarization [13]. Treatment of murine peritoneal macrophages with wortmannin, a specific inhibitor of PI3K, was found to augment LPS-induced NOS2 expression [14]. At the genetic level, inhibiting the PI3K/Akt pathway can significantly increase the level of the inflammatory factors TNFα and IL-6 in the plasma of endotoxemia mice [15]. However, the specific mechanism regulating the M1/M2 polarization of macrophages remains elusive.

TLR4, cytokines, and chemokines, as well as activated fragment crystallizable (Fc) receptors, can activate the PI3K/Akt signaling pathway [11, 16, 17]. PI3K is an intracellular phosphatidylinositol kinase. Mammals express four class I catalytic subunits, namely p110α, β, δ, and γ. These subunits are encoded by the genes PIK3CA, PIK3CB, PIK3CD, and PIK3CG, respectively. Studies have shown that overexpression and inhibition of PIK3CD can affect the level of p-Akt downstream of it [18]. While p110α and p110β are widely expressed, p110δ and p110γ are mainly expressed in immune cells [19].

IBD is a cluster of chronic, recurrent inflammatory diseases, such as Crohn's disease and ulcerative colitis [20]. Intestinal immune cells, especially colonic macrophages, are pivotal in the pathogenesis of IBD. Studies have shown that the phenotypic transformation of a large number of macrophages in the colon is closely related to IBD [21, 22]. M1 macrophages and pro-inflammatory cytokines exacerbate the progression of IBD, whereas M2 macrophages facilitate tissue repair and release anti-inflammatory cytokines, thereby mitigating IBD symptoms [23]. Because the abnormal polarization of macrophages occurs during the occurrence and progression of IBD, it can directly affect the outcome of IBD [24, 25], so regulating macrophage polarization may be a potential treatment strategy for IBD. As a first step toward that therapeutic development goal, an in-depth study to determine the exact mechanism of macrophage polarization is necessary.

FAM76B has been reported to contain a tract of poly-his domain and is located in the nuclear speckle body [26]. Many proteins containing poly-his have been shown to be associated with the functions of DNA and RNA. The nuclear speckle body is a substructure of the nucleus that can assemble and preserve the RNA splicing complex. Its structure and function are closely related to the transcriptional state of the cells. The high conservation of FAM76B across diverse species implies that it may have crucial biological functions; however, these functions remain unclear.

Our previous results found that FAM76B expression was significantly increased in human lymph nodes and spleen [27]. Recent results in our lab also indicated that FAM76B showed high-level expression in the immune system of zebrafish [28]. These results suggested its potential involvement in immune regulation. The recent research by Wang et al. in our laboratory demonstrated that FAM76B could regulate neuroinflammation by influencing the translocation of the RNA-binding protein hnRNPA2B1 [29]. However, further in-depth studies are urgently needed to explore whether FAM76B could regulate other aspects of the inflammation process. In this study, we, for the first time, revealed in vitro that FAM76B regulated PI3K/Akt/NF-κB-mediated M1 macrophage polarization by influencing the stability of PIK3CD mRNA. In vivo*,* FAM76B was demonstrated to protect against IBD by inhibiting M1 macrophage polarization through the PI3K/Akt/NF-κB pathway. These results provide a basis for developing potential FAM76B-based therapeutic strategies to treat some inflammation-related diseases.

## Materials and methods

### Cell culture and induction of macrophage polarization

Our laboratory successfully established stable *Fam76b* knockout U937 cell lines and control cell lines through lentivirus infection at an early stage [29]. After detection without mycoplasma contamination, two types of U937 cells were cultured in a 1640 cell culture medium with 10% fetal bovine serum (FBS). To restore FAM76B in *Fam76b* knockout U937 cells, viral infection was employed by infecting *Fam76b* knockout U937 cells with either the LV-CMV-h*Fam76b*-EF1-GFP lentivirus or the control LV-CMV-MCS-EF1-GFP lentivirus for 72 h. The U937 mentioned above cells were cultured with 10 ng/ml PMA for 48 h to induce macrophages [30]. Subsequently, the cells were exposed to a combination of 10 ng/ml LPS (Sigma-Aldrich, St. Louis, MO, USA) and 2 ng/ml IFN γ for 24 h to induce M1 macrophages. Alternatively, they were subjected to 5 ng/ml IL-4 and 10 ng/ml IL-13 for 24 h to induce M2 macrophages. RAW264.7 cells were cultured in DMEM (Gibco, Carlsbad, CA, USA) with 10% FBS. When the cells had grown to about 70%, they were treated with 100 ng/ml LPS and 10 ng/ml IFN γ for 24 h to induce M1 macrophages or with 10 ng/ml IL-4 and 10 ng/ml IL-13 for 24 h to induce M2 macrophages. IFN γ, IL-4, and IL-13 used to induce the cells were all purchased from Sino Biological Inc. (Beijing, China).

### Isolation and induction of bone marrow macrophages and peritoneal macrophages

The cells in the femur and tibia of wild-type mice and *Fam76b* knockout mice were rinsed with DMEM containing 3% FBS. Non-monocytes were then allowed to adhere. Monocytes in the culture supernatant were centrifuged at 350 g for 4 min at 4 °C and plated in DMEM supplemented with 10 ng/ml M-CSF. After 6 d of culture, the cells were successfully induced into bone marrow macrophages (BMMs). To restore FAM76B in BMMs from *Fam76b* knockout mice, BMMs from *Fam76b* knockout mice were infected with either the LV-CMV-m*Fam76b*-EF1-GFP lentivirus or the control LV-CMV-MCS-EF1-GFP lentivirus for 72 h.

The peritoneal cavity was washed with peritoneal wash (1 × D-hanks with 3% newborn calf serum [NCS]) to obtain peritoneal macrophages (PEMs), which were cultured in DMEM. After 2 d of culture, these cells were used for subsequent experiments. To induce the polarization of BMMs and PEMs, the cells were treated with 100 ng/ml LPS and 10 ng/ml IFN γ for 24 h to induce M1 macrophages or with 10 ng/ml IL-4 and 10 ng/ml IL-13 for 24 h to induce M2 macrophages. M-CSF, IFN γ, IL-4, and IL-13 used to induce the cells were all purchased from Sino Biological Inc. (Beijing, China).

### RNA-seq

To induce M1 macrophage, *Fam76b* knockout U937 cell lines and control cell lines were cultured with 10 ng/ml PMA for 48 h to induce macrophages, followed by the stimulation of 10 ng/ml LPS and 2 ng/ml IFNγ. The obtained control U937 cells with M1 phenotype and *Fam76b* knockout U937 cells with M1 phenotype were used for RNA-seq. Beijing Baimaike Biotechnology Co., Ltd. (BMK, Beijing, China) performed RNA preparation, library construction, and sequencing using a BMK instrument to generate high-quality RNA sequencing data. The expectation–maximization (RSEM) method was employed for gene expression quantification. The NOISeq method was used to screen for differentially expressed genes between the control U937 cells with M1 phenotype and *Fam76b* knockout U937 cells with M1 phenotype, and hierarchical clustering was performed using Cluster. The screening criteria for identifying deferentially expressed genes are defined as follows: *p* < 0.05 and the absolute value of log2 Ratio ≥ 1.

### qPCR

Cells were subjected to total RNA isolation using TRIzol reagent (Invitrogen, Carlsbad, CA, USA). Subsequently, cDNA synthesis was carried out using the PrimeScript RT reagent Kit (TaKaRa, Dalian, Liaoning, China). Quantitative PCR (qPCR) experiments were conducted using a PCR Kit (SYBR green) (QIAGEN, Hilden, Germany) on an ABI 7900HT Fast Real-Time PCR system (Applied Biosystems, Foster, CA, USA). Gene expression was normalized to GAPDH. The primer sequences used for qPCR are provided in Table S1.

### Immunofluorescence staining

Cells were subjected to a fixation using 4% paraformaldehyde (PFA) for 15 min. Subsequently, cells were treated with 0.5% Triton X-100 for 25 min for permeabilization. Then, a blocking step was carried out using 3% bovine serum albumin (BSA) (diluted in 1 × PBS) for 30 min. Next, cells were incubated with anti-NOS2 (1:200, Proteintech, Chicago, IL, USA) or anti-CD68 (1:200, SAB, Nanjing, China) overnight. The secondary antibody was stained with goat anti-rabbit IgG Dylight 488 (1:500, Abbkine, Wuhan, China) away from light for 1 h. Nuclei were stained with DAPI. The acquisition of images was carried out using a Zeiss-Axio Imager M2 microscope (Zeiss, Oberkochen, Germany).

### Western blot

Cells and tissue samples were lysed using RIPA buffer and quantitated using a bicinchoninic acid assay (BCA). Protein extracts were separated using SDS-PAGE and transferred to a polyvinylidene difluoride (PVDF) membrane with a transfer apparatus following the manufacturer’s protocols. Then the membrane was incubated with antibodies against FAM76B (1:500; monoclonal antibody, mouse; Homemade); NOS2 (1:1000), Arg-1 (1:500), p110δ (1:500), p-Akt (Ser473,1:1000), Akt (1:500) (all purchased from Proteintech, Chicago, IL, USA); p-NF-κB p65 (Ser536, 1:500), NF-κB p65(1:1000) (both purchased from Cell Signaling Technology, Danvers, MA, USA); or β-actin (rabbit polyclonal, 1:1000, Santa Cruz, Dallas, TX, USA) at 4 °C overnight. Subsequently, the membrane was placed in horseradish peroxidase (HRP)-labeled Goat IgG antibody (anti-rabbit or anti-mouse, 1:10,000) (from Abbkine, Wuhan, China) and incubated for 1 h with slow shaking in a shaker. Then, the PVDF membrane was immersed in a photoluminescent solution for imaging purposes. The protein bands were analyzed using ImageJ software v1.8.0.

### RIP-seq

A stable U937 cell line was established by infection with *Fam76b*-Strep tag II lentiviruses by our laboratory at an early stage. Then, these cells were subjected to RIP experiments utilizing a Magna RIP™ RNA-Binding Protein Immunoprecipitation Kit (Millipore, Billerica, MA, USA). Cells were harvested and incubated for 3–4 h at 4 °C with Strep-Tactin beads (QIAGEN, Düsseldorf, Germany). RIP and Input products were sent to RIBO (Guangzhou, China). After passing the quality control test, RIP-seq was performed on the Illumina platform. The RIP-seq report was provided by RIBO.

### RIP experiment

A Magna RIP™ RNA-Binding Protein Immunoprecipitation Kit (Millipore, Billerica, MA, USA) was employed following the manufacturer's recommendations. Briefly, a stable U937 cell line was established by infection with *Fam76b*-Strep tag II or control *Fam76b*-expressing lentiviruses by the laboratory at an early stage. Then, cells were harvested and incubated for 3–4 h at 4 °C with Strep-Tactin beads (QIAGEN, Düsseldorf, Germany). HEK293 cells were transfected with a eukaryotic expression plasmid of overexpressed FAM76B with a Flag tag (pcDNA/CMV-*Fam76b*-flag). Cells were harvested and incubated for 3–4 h at 4 °C with magnetic beads coated with Flag antibody (Proteintech, Chicago, IL, USA) or IgG antibody.

Then, the beads mentioned above were washed six times, after which the protein was digested with proteinase K. RNA from immune precipitates, and input was extracted using a phenol–chloroform-isoamyl alcohol reagent. qPCR was used to quantify PIK3CD mRNA.

### The detection of the interaction between FAM76B and PIK3CD mRNA

To detect the interaction between FAM76B and PIK3CD mRNA using the BASU-dCasRx system, six pairs of primers for sgRNA targeting PIK3CD mRNA (Table S2) were designed using the Cas13 website (https://cas13design.nygenome.org/); mCherry sgRNA was used as a control in the experiment. Then, the primers were annealed and ligated into the CasRx-SgRNA plasmid, transfecting HEK293 cells. The targeting of the six pairs of sgRNA to PIK3CD mRNA was detected by qPCR. Next, the selected sgRNA with good targeting was inserted into the BASU-dCasRx-SgRNA plasmid, using mCherry sgRNA as a control group. HEK293 cells were transfected with the constructed plasmids for 48 h followed by treatment with biotin for 15 min. Subsequently, the cells were harvested and incubated for 2 h at 4 °C with Strep-Tactin beads (QIAGEN, Düsseldorf, Germany), and FAM76B was detected by Western blot.

### Mice

Customized homozygous *Fam76b* knockout mice were created by gene trap mutagenesis techniques at the Texas A&M Institute for Genomic Medicine (College Station, TX, USA). The description of the production of *Fam76b* knockout mice was published in the previous study in our lab[29]. Two fertile male chimeras were obtained and then bred with C57BL/6 mice. After breeding, the mouse tail genome was extracted. PCR was used for the genotype of the offspring identification. Mouse genotype was carried out according to the methods described in the previous report [29]. The electrophoresis results of the PCR products are shown in Fig. S1. The primers used for mouse genotype identification are shown in Table S3.

### DSS-induced colitis

All experiments were conducted using *Fam76b* knockout mice; wild-type mice matched in age and sex were used as control. The mice were kept on a 12/12 h light/dark cycle with unrestricted access to water and food. WT and *Fam76b* knockout mice were randomly assigned and received 2.5% DSS (MP Biomedicals, Irvine, CA, USA, M.W. 36,000–50,000 kDa) for 7 day. The Disease Activity Index (DAI) was calculated based on the consistency of fecal, fecal bleeding and degree of weight loss according to the scoring criteria utilized in the literature [24]. All parameters were evaluated and recorded daily from day 0 to 7. On day 8, the mice were sacrificed, and the entire colon was promptly extracted for assessment of colon length and other indicators.

### Hematoxylin & eosin (H&E), histopathological score and immunohistochemical staining

Colon tissue from mice was immersed in 4% PFA for 48 h. Then, the tissues were subjected to paraffin embedding and sliced into sections measuring 5 mm. Subsequently, H&E staining was carried out according to our previous staining procedure [31]. The acquisition of images was carried out using a Nikon Eclipse E800 microscope (Nikon, Tokyo, Japan).

Three fields of the colon section were randomly selected for histopathological scores. Histopathological scores were performed according to the following scoring criteria [32]: normal colon tissue is recorded as 0 points; mild mucosal inflammation with partial monocytes infiltration is recorded as 1 point; the increased level of mucosal inflammation with increased infiltrating cells and damaged cryptic glands and epithelium is recorded as 2 points; extensively infiltrated cells in the mucosa and submucosa with crypt abscesses and epithelial cell destruction is recorded as 3 points; a large number of cells infiltrated the tissue with the crypt completely disappeared is recorded as 4 points.

Paraffin-embedded tissue sections were sliced into a thickness of 5 mm for immunohistochemical staining. High-pressure repair was carried out first using a citrate buffer. Sections were immersed in a solution of 3% H_2_O_2_ in methanol for 10 min. Subsequently, the sections were rinsed with distilled water and then blocked using 5% goat serum for 1 h. Sections were incubated with NOS2 antibody (1:200) (Proteintech, Chicago, IL, USA) overnight. Biotinylated secondary antibodies were amplified with avidin–biotin substrate and then color-developed in DAB chromogen. The images were taken with a Nikon Eclipse E800 microscope (Nikon, Tokyo, Japan) and were analyzed using Image J software (version: v1.8.0) to obtain an integrated option density (IOD) for quantitative staining results.

### Immunofluorescence double-staining

The paraffin-embedded colon tissues from wild-type and *Fam76b* knockout mice induced by DSS were sectioned. Slices need to be performed the following procedures: dewaxing to water, antigen repair, inactivation of endogenous peroxidase, and the block of BSA. Next, primary antibody incubation was performed using: F4/80 (rabbit polyclonal, 1:1000), anti-NOS2 (rabbit polyclonal, 1:500) (both purchased from Servicebio Co. Ltd., Wuhan, China), and anti-p110δ (rabbit polyclonal, 1:500, Proteintech, Chicago, IL, USA). Then, the corresponding HRP-labeled secondary antibody was added to the tissue area of the slides and incubated at room temperature for 50 min. After that, Tyramide Signal Amplification (TSA) staining working solution (Servicebio Co., Ltd., Wuhan, China) was added to the tissue area of the slides and incubated for 10 min in the dark. After the second run, the nucleus was stained with a DAPI staining work solution. Finally, representative images of staining were captured using a Zeiss Imager M2 microscope. The number of F4/80^+^NOS2^+^ cells or F4/80^+^p110δ^+^ cells were counted and analyzed statistically.

### Statistical analysis

A two-tailed Student’s *t*-test was employed to compare the two groups if both groups were normally distributed, while the rank-sum test was used if at least one group was not normally distributed. For the comparison of three or more groups, one-way ANOVA was utilized, followed by multiple comparisons. Statistical analysis of the collected data was performed using GraphPad Prism 8.0 software. The results are presented as means ± SD. In all figures, *p < 0.05 indicates that the difference was statistically significant.

## Results

### FAM76B inhibits the polarization of M1 macrophages in vitro

**Fig. 1:**
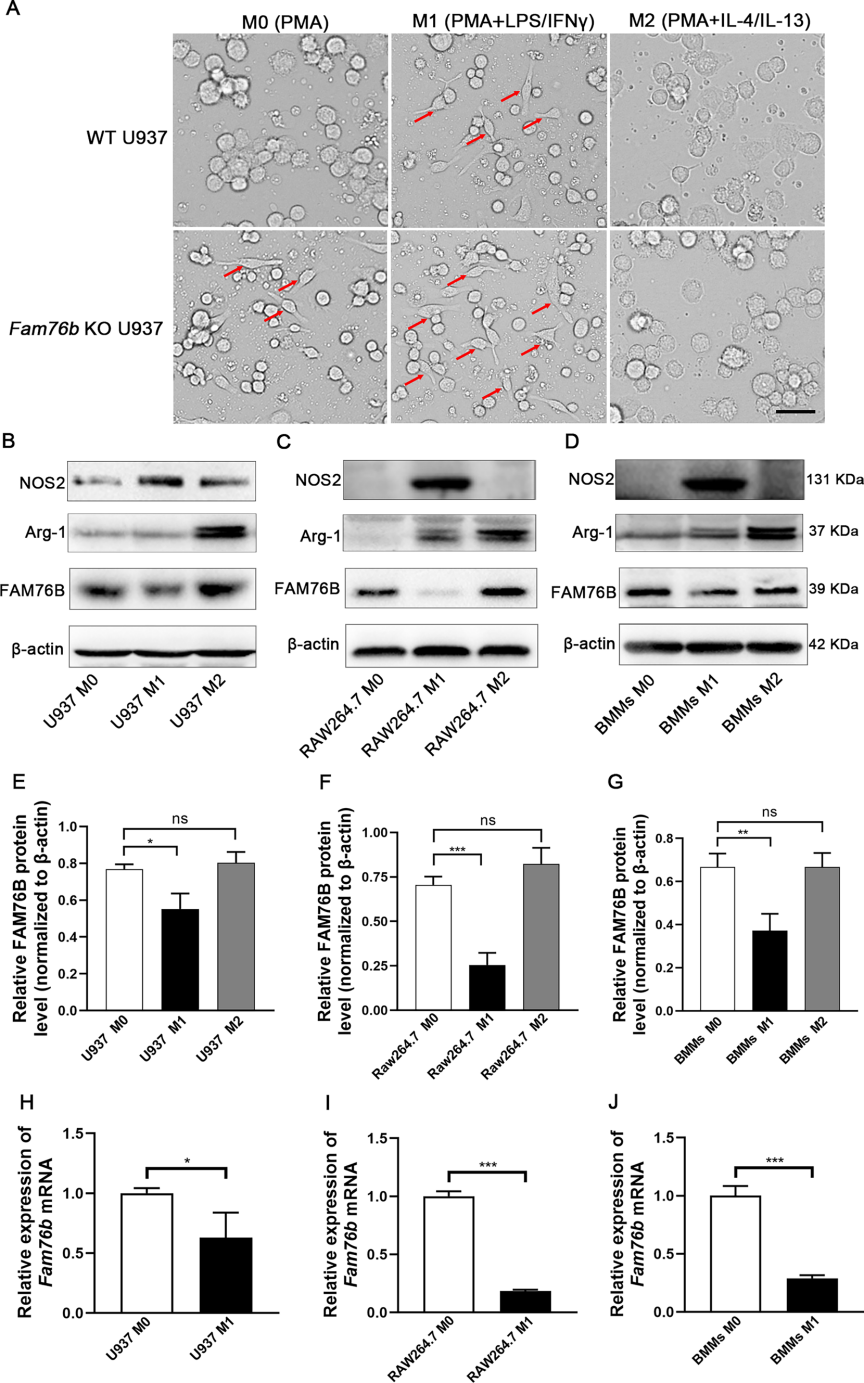
The effect of FAM76B on macrophage polarization. **A** The cell morphology of PMA-treated wild-type (WT) and *Fam76b* knockout (KO) U937 cells in the absence or presence of LPS/IFNγ or IL-4/IL-13. The long spindle cells indicated by the red arrows are M1-like macrophages, Scale bar, 50 μm. The protein level of FAM76B, NOS2, and Arg-1 was detected by Western blot in M0, M1, and M2 U937 cells (**B**), RAW264.7 cells (**C**), and BMMs (**D)**. **E–G** The quantification results of FAM76B protein bands in Fig. 1**B**–**D**. The mRNA level of FAM76B in M0 and M1 U937 cells (**H**), RAW264.7 cells (**I**) and BMMs (**J**) was detected by qPCR. The data are presented as means ± SD, *n* = 3, *P < 0.05, **P < 0.01, and ***P < 0.001. *n.s*., not significant

First, we verified the successful deletion of FAM76B in *Fam76b* knockout U937 cells by Western blot (Fig. S2). Morphologically, we found long spindle M1 macrophages in *Fam76b* knockout U937 cells treated with PMA (phorbol 12-myristate 13-acetate) vs. wild-type U937 cells (Fig. 1A). When PMA-treated *Fam76b* knockout cells were further stimulated by LPS/IFNγ for 24 h, the number of M1 macrophages was markedly increased compared to wild-type U937 cells (Fig. 1A). However, there was no observable difference in the morphology of the M2 macrophages between the PMA-treated wild-type and *Fam76b* knockout U937 cells in the presence of IL-4/IL-13 (Fig. 1A), suggesting that FAM76B did not affect M2 polarization. We next examined the level of FAM76B protein in macrophages with different phenotypes. To confirm that M1 or M2 macrophage was successfully induced, we first detected the related markers of M1 or M2 macrophage. The results showed that the protein level of NOS2 in U937 (Fig. 1B), RAW264.7 (Fig. 1C), and BMMs (Fig. 1D) was noticeably increased after LPS/IFN γ induction, indicating that M1 macrophages were successfully induced. After stimulation with IL-4/IL13, there was a significant increase in Arg-1 expression in three types of macrophages (Fig. 1B–D), indicating that these cells were successfully induced into M2 macrophage phenotypes. Then, the protein levels of FAM76B were detected in the cells above. The results indicated that the level of FAM76B protein in M1 macrophages induced from U937 cells (Fig. 1B), RAW264.7 cells (Fig. 1C), and BMMs (Fig. 1D) was found to be significantly decreased compared to the corresponding M0 macrophages, while the protein levels of FAM76B in M2 macrophages induced from the corresponding cell groups were not significantly changed (Fig. 1B–D). The quantitative results of FAM76B protein levels in each group are shown in Fig. 1E–G. The qPCR analysis revealed that the mRNA levels of FAM76B in M1 macrophages induced from U937 cells (Fig. 1H), RAW264.7 cells (Fig. 1I), and BMMs (Fig. 1J) were significantly decreased compared to the corresponding M0 macrophages. The data above suggested that FAM76B has to be downregulated to allow M1 polarization.

**Fig. 2:**
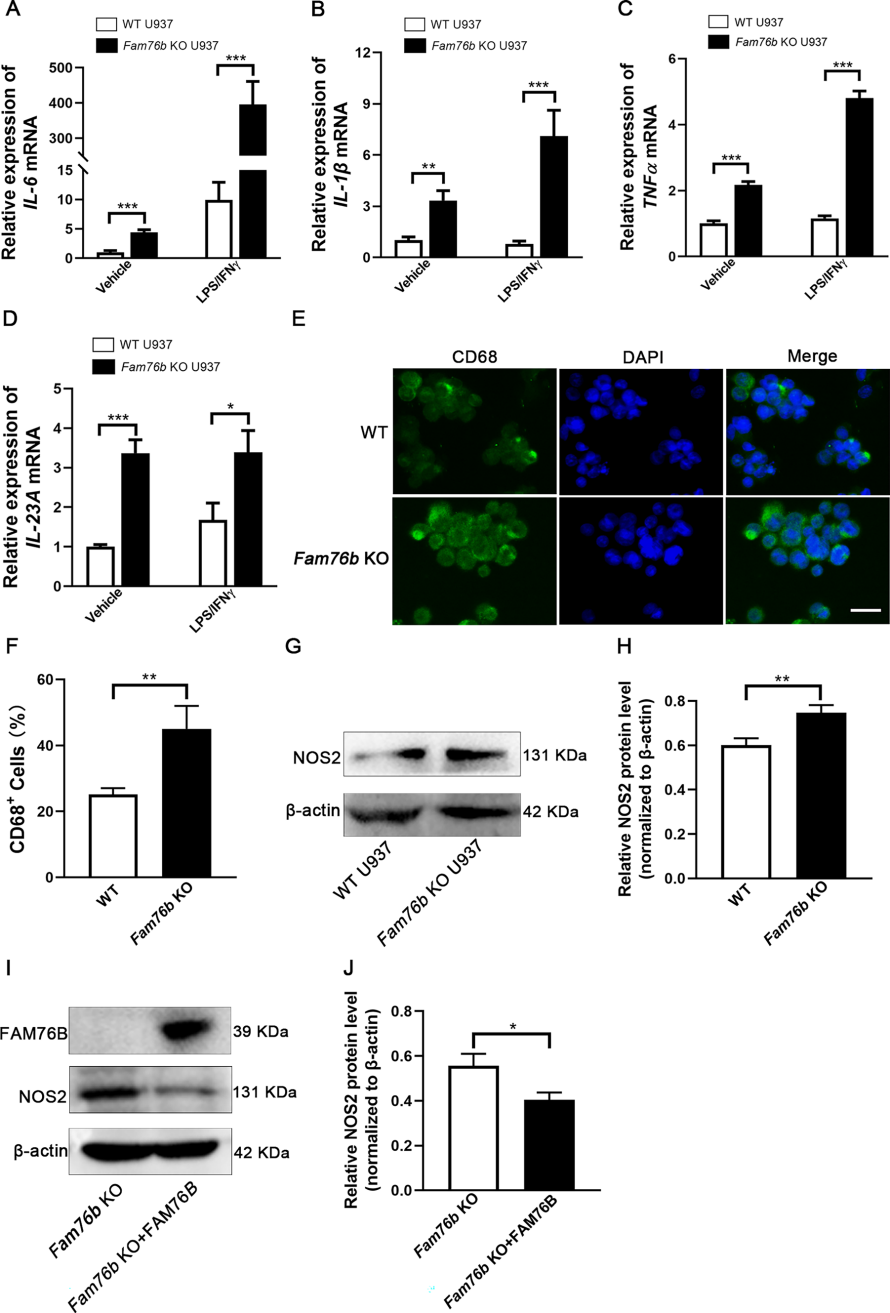
*Fam76b* knockout promotes M1 macrophage polarization in U937 cells. **A**–**D** The expression of IL-6 (**A**), IL-1β (**B**), TNFα (**C**), and IL-23A (**D**) were detected by qPCR in wild-type and *Fam76b* knockout U937 cells stimulated with vehicle (PBS) or LPS/IFNγ for 24 h after stimulation with PMA for 48 h, *n* = 3. **E** Immunofluorescence staining of CD68 was detected in wild-type and *Fam76b* knockout U937 cells treated with PMA for 48 h, Scale bar, 100 μm. **F** The quantification result of CD68^+^ cell number in Fig. 2**E**. **G** The protein level of NOS2 in wild-type U937 and *Fam76b* knockout U937 cells treated with PMA for 48 h was measured by Western blot. **H** The quantification of NOS2 protein bands in Fig. 2**G**. **I** The expression of FAM76B, NOS2, and β-actin was assessed using Western blot in *Fam76b* knockout U937 cells and *Fam76b* knockout U937 cells supplemented with FAM76B. **J** The quantification of NOS2 protein bands in Fig. 2I. The data are presented as means ± SD, *n* = 3, *P < 0.05, **P < 0.01, and ***P < 0.001

In order to further confirm the influence of FAM76B on M1 macrophage polarization, the expression of the marker genes of M1 macrophages was detected by qPCR, immunofluorescence, or Western blot in PMA-treated wild-type and *Fam76b* knockout U937 cells. The qPCR analysis revealed that *Fam76b* knockout could promote the expression of IL-6 (Fig. 2A), IL-1β (Fig. 2B), TNFα (Fig. 2C), and IL-23A (Fig. 2D). To investigate further the effect of FAM76B on the protein levels of macrophage polarization-related genes, we employed cell immunofluorescence to assess the effect of FAM76B on the protein levels of the macrophage marker gene CD68 and utilized Western blot to assess the effect of FAM76B on the protein levels of M1macrophage marker gene NOS2. Immunofluorescence results showed that CD68 expression in *Fam76b* knockout U937 cells was markedly elevated in comparison to that in wild-type U937 cells (Fig. 2E). The quantification of CD68^+^ cell number (Fig. 2E) is shown in Fig. 2F. Western blot analysis demonstrated that the protein level of NOS2 was increased in *Fam76b* knockout U937 cells compared to wild-type U937 cells (Fig. 2G). The quantification results of NOS2 from Western blot (Fig. 2G) are shown in Fig. 2H. To further validate the role of FAM76B in regulating M1 macrophage polarization, we overexpressed FAM76B in *Fam76b* knockout U937 cells to detect the effect of FAM76B on the protein level of NOS2. Western blot analysis demonstrated that the successful rescue of FAM76B in *Fam76b* knockout U937 cells significantly down-regulated the expression of NOS2 (Fig. 2I). The quantification results of NOS2 from Western blot (Fig. 2I) are shown in Fig. 2J. The elevated expression of marker genes of M1 macrophages in *Fam76b* knockout U937 cells further demonstrated that FAM76B deletion promoted M1 polarization.

**Fig. 3:**
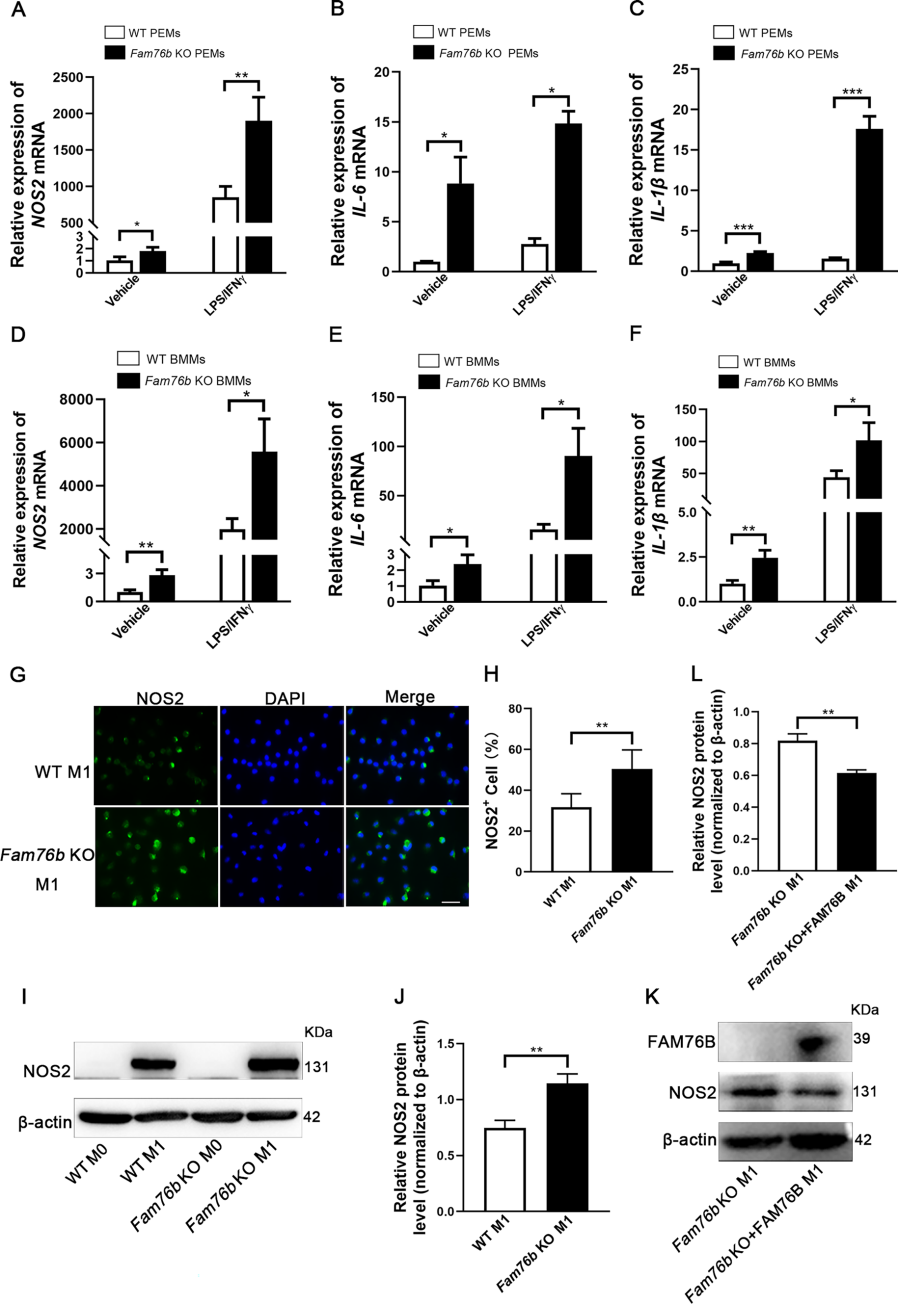
*Fam76b* knockout promotes M1 macrophage polarization in PEMs and BMMs. **A**–**C** The expression of NOS2 (**A**), IL-6 (**B**), and IL-1β (**C**) were detected by qPCR in PEMs stimulated with vehicle (PBS) or LPS/IFNγ for 24 h from wild-type and *Fam76b* knockout mice, *n* = 3. **D**–**F** The expression of NOS2 (**D**), IL-6 (**E**), and IL-1β (**F**) were detected by qPCR in BMMs stimulated with vehicle (PBS) or LPS/IFNγ for 24 h from wild-type and *Fam76b* knockout mice, *n* = 3. **G** Immunofluorescence staining of NOS2 in BMMs stimulated with LPS/IFNγ for 24 h from wild-type and *Fam76b* knockout mice, Scale bar, 50 μm. **H** The quantification result of NOS2^+^ cell number in Fig. 3**G**, n = 5. **I** The protein level of NOS2 in BMMs stimulated with LPS/IFNγ for 24 h (or in unstimulated BMMs) from wild-type and *Fam76b* knockout mice was detected by Western blot. **J** The quantification of NOS2 protein bands in Fig. 3**I**. **K** The expression of FAM76B, NOS2, and β-actin was assessed using Western blot in BMMs from *Fam76b* knockout mice and BMMs from *Fam76b* knockout mice supplemented with FAM76B. **L** The quantification of NOS2 protein bands in Fig. 3**K**, n = 3. *P < 0.05, **P < 0.01, and ***P < 0.001

Next, to explore whether FAM76B could inhibit the polarization of macrophages derived from mouse PEMs and BMMs, the marker genes of M1 macrophages were detected in PEMs and BMMs from wild-type and *Fam76b* knockout mice. The qPCR analysis revealed that the expression of M1-associated genes, including NOS2 (Fig. 3A), IL-6 (Fig. 3B), and IL-1β (Fig. 3C), in PEMs from *Fam76b* knockout mice were markedly higher than those in PEMs from wild-type mice. Similar results were obtained in BMMs (Fig. 3D–F). The expression of NOS2 in BMMs from wild-type and *Fam76b* knockout mice was also detected by immunofluorescence and Western blot. Immunofluorescence demonstrated that the protein level of NOS2 in M1 phenotype BMMs from *Fam76b* knockout mice was markedly higher than that in wild-type mice (Fig. 3G). The quantification of NOS2^+^ cells is shown in Fig. 3H. Similar results were also confirmed by Western blot (Fig. 3I), and quantification of the NOS2 protein bands is shown in Fig. 3J. Next, we overexpressed FAM76B in BMMs from *Fam76b* knockout mice to further examine the effect of FAM76B on M1 macrophage polarization. Western blot results showed that FAM76B supplementation in BMMs from *Fam76b* knockout mice could significantly reduce the protein level of NOS2 (Fig. 3K). The quantification results of NOS2 from Western blot (Fig. 3K) are shown in Fig. 3L. These results further confirmed that FAM76B could inhibit M1 macrophage polarization.

### FAM76B inhibits PI3K/Akt/NF-κB-mediated M1 macrophage polarization by affecting the stability of PIK3CD mRNA

**Fig. 4:**
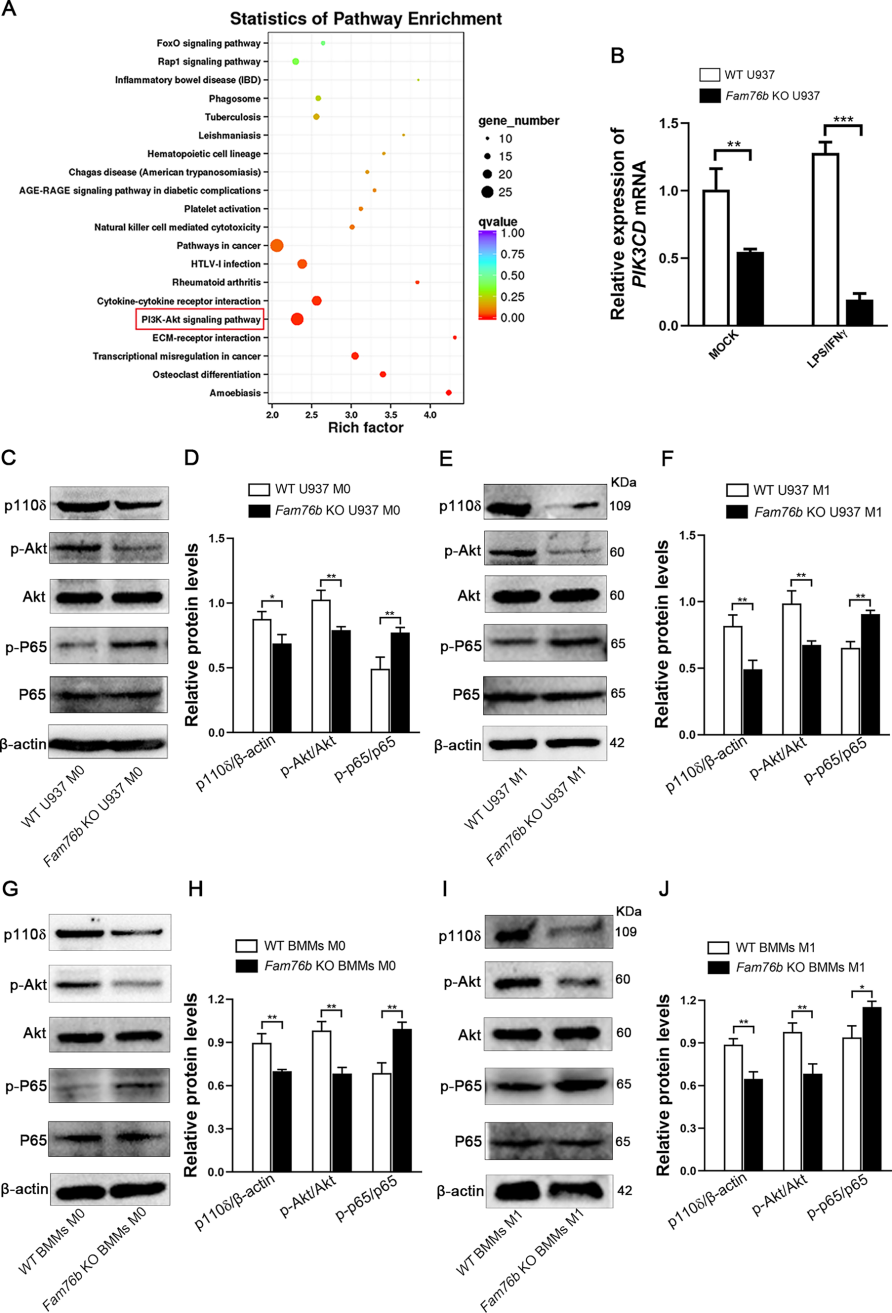
FAM76B modulates the PI3K/Akt/NF-κB pathway by affecting the level of PIK3CD mRNA. **A** Wild-type and *Fam76b* knockout U937 cells were induced with LPS and IFNγ for 24 h after stimulation with PMA for 48 h. Total RNA was extracted and used to perform RNA-seq experiments. A KEGG enrichment analysis of the signaling pathways from the RNA-seq data was performed. **B** The expression of PIK3CD was examined using qPCR in wild-type and *Fam76b* knockout U937 cells with the M0 or M1 phenotype. **C** The expression of p110δ, p-Akt, Akt, p-p65, p65, and β-actin were measured using Western blot in wild-type and *Fam76b* knockout U937 cells with the M0 phenotype. **D** Quantification results of each protein band in Fig. 4**C**. **E** The protein levels of p110δ, p-Akt, Akt, p-p65, p65, and β-actin were measured using Western blot in wild-type and *Fam76b* knockout U937 cells with M1 phenotype. **F** Quantification results of each protein band in Fig. 4**E**. **G** The expression of p110δ, p-Akt, Akt, p-p65, p65, and β-actin were measured using Western blot in BMMs from wild-type and *Fam76b* knockout mice. (**H**) The quantification of protein bands in Fig. 4**G**. **I** The protein levels of p110δ, p-Akt, Akt, p-p65, p65, and β-actin were measured using Western blot in BMMs stimulated with LPS/IFNγ for 24 h from wild-type and *Fam76b* knockout mice. **J** Quantification results of each protein band in Fig. 4**I**. The data are presented as means ± SD, *n* = 3, *P < 0.05, **P < 0.01, ***P < 0.001

To investigate further the potential mechanism of FAM76B inhibiting M1 macrophage polarization, RNA-seq was carried out on wild-type and *Fam76b* knockout U937 cells with the M1 phenotype. The result of RNA-seq revealed that the PI3K/Akt pathway was highly enriched based on an analysis of KEGG (Kyoto Encyclopedia of Genes and Genomes) pathways (Fig. 4A). Previous studies had demonstrated a significant correlation between the PI3K/Akt pathway and the regulation of macrophage polarization [13, 15]. In addition, the mRNA level of PIK3CD encoding the catalytic subunit p110δ of PI3K was found to be significantly decreased in *Fam76b* knockout U937 cells compared to wild-type U937 cells based on the analysis of RNA-seq results. Next, this decreased PIK3CD mRNA level was further verified in U937 cells by qPCR (Fig. 4B). Moreover, RNA-seq results showed that *Fam76b* knockout significantly increased the mRNA level of NF-κB which has crosstalk with the PI3K/Akt signaling pathway (Fig. S3). Given the presence of crosstalk between the PI3K/Akt and NF-κB signal pathways, we next detected the protein level of p110δ, p-Akt, and p-NF-κB p65 through Western blot. The protein levels of p110δ and p-Akt were found to be markedly decreased. In contrast, the protein level of p-NF-κB p65 was significantly increased in M0 and M1 macrophages induced from *Fam76b* knockout U937 cells (Fig. 4C, E). The quantification of protein bands is shown in Fig. 4D and F. The protein levels of p110δ and p-Akt were also found to be significantly decreased. In contrast, the protein level of p-NF-κB p65 was markedly increased in M0 macrophages induced from *Fam76b* knockout BMMs, as assessed by Western blot (Fig. 4G). The quantification of the protein bands from this Western blot (Fig. 4G) is shown in Fig. 4H. Similar results were also confirmed by determining the expression of p110δ, p-Akt, and p-NF-κB p65 in M1 macrophages induced from *Fam76b* knockout BMMs through Western blot (Fig. 4I). The quantification of the protein bands from this Western blot (Fig. 4I) is shown in Fig. 4J.

**Fig. 5:**
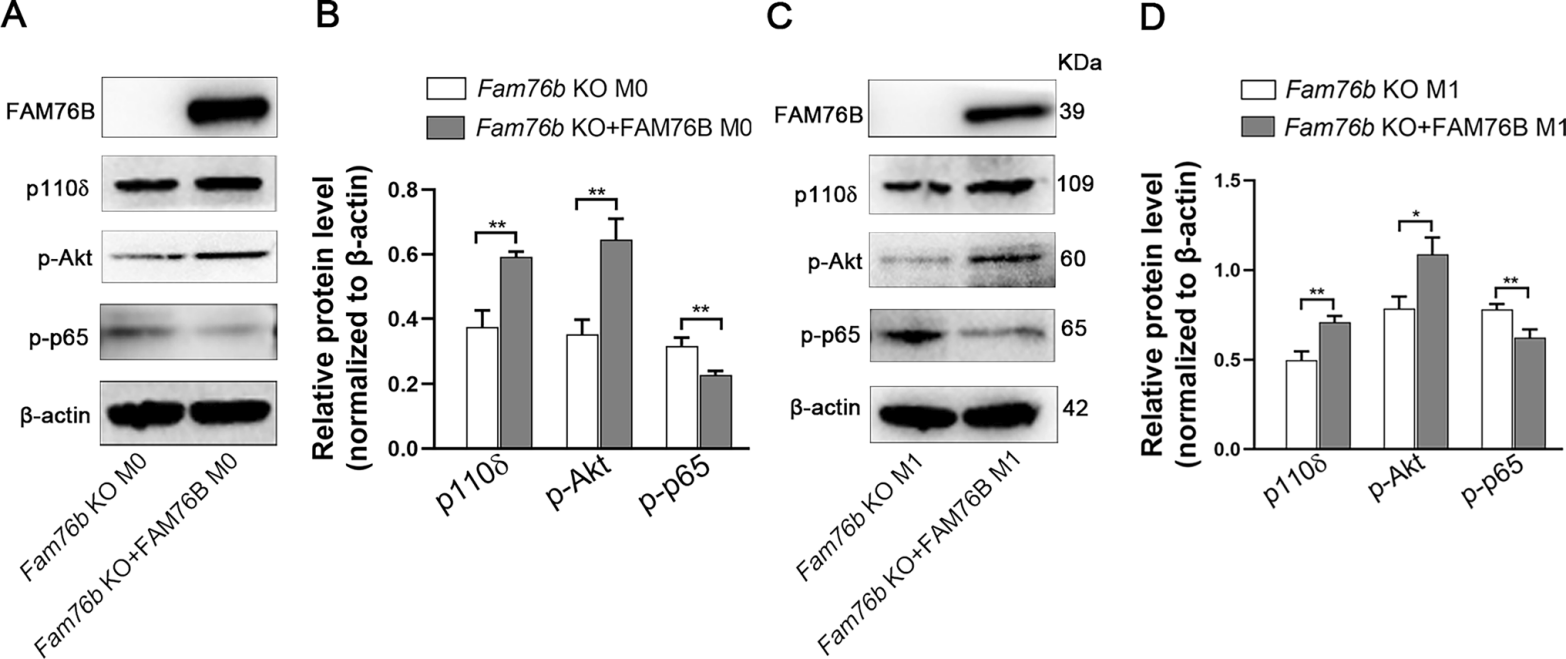
FAM76B regulates M1 macrophage polarization via the PI3K/Akt/NF-κB pathway. **A** The expression of FAM76B, p110δ, p-Akt, p-p65, and β-actin was assessed using Western blot in *Fam76b* knockout U937 cells and *Fam76b* knockout U937 cells supplemented with FAM76B. **B** The quantification result of each protein band is in Fig. 5**A**. **C** The expression of FAM76B, p110δ, p-Akt, p-p65, and β-actin was assessed using Western blot in M1 *Fam76b* knockout U937 cells and M1 *Fam76b* knockout U937 cells supplemented with FAM76B. **D** The quantification result of each protein band is in Fig. 5**C**. The data are presented as means ± SD, n = 3, *P < 0.05, **P < 0.01

To further clarify the role of FAM76B in modulating the PI3K/Akt/NF-κB signaling axis, we overexpressed FAM76B in M0 *Fam76b* knockout U937 cells. Western blot analysis demonstrated that rescuing FAM76B in M0 *Fam76b* knockout U937 cells could significantly increase the expression of p110δ and p-Akt and decrease the expression of p-NF-κB p65 (Fig. 5A). The quantification result of p110δ, p-Akt, and p-NF-κB p65 from Western blot is shown in Fig. 5B. Similar results were also obtained in M1 *Fam76b* knockout U937 cells supplemented with FAM76B (Fig. 5C). The quantification result of p110δ, p-Akt, and p-NF-κB p65 (Fig. 5C) is shown in Fig. 5D. The results indicate that FAM76B regulates M1 macrophage polarization by modulating the PI3K/Akt/NF-κB pathway.

**Fig. 6:**
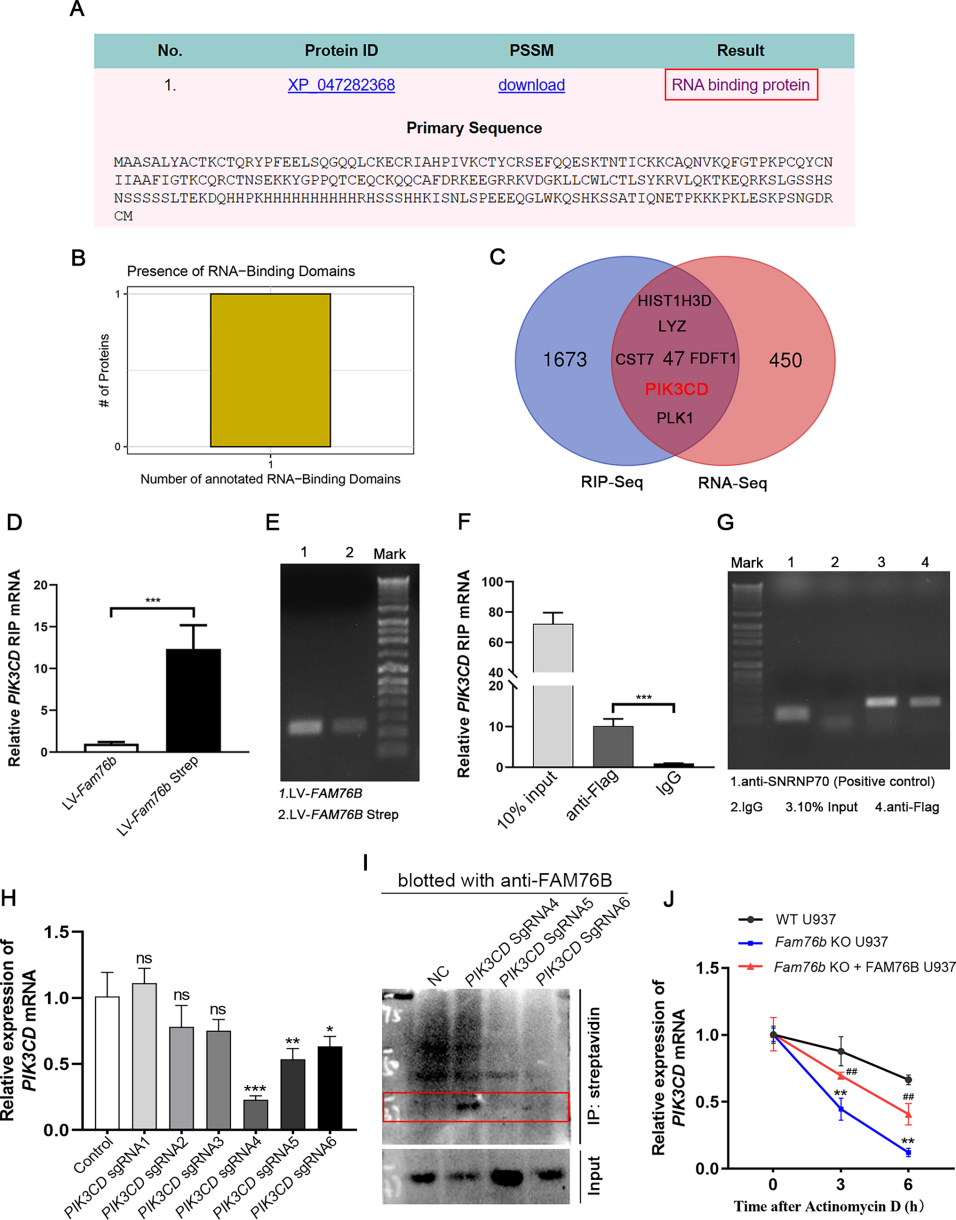
FAM76B binds to PIK3CD mRNA, and *Fam76b* knockout decreases the stability of PIK3CD mRNA. **A** iDRBP_MMC predictions showed that FAM76B is an RNA-binding protein. **B** catRAPID predictions showed that FAM76B has an RNA-binding domain in its protein sequence. **C** Venn analysis of the mRNAs that can bind with FAM76B (screened from RIP-seq) and that are differentially expressed (as determined by RNA-seq). **D** U937 cells were infected by *Fam76b*-Strep-tag- (LV-*Fam76b*-strep) and *Fam76b*-tag- (LV-*Fam76b*) expressing lentiviruses, then the stable cell line selection was carried out. The qPCR analysis of RIP assay results for FAM76B binding to PIK3CD mRNA using LV-*Fam76b*-Strep and LV-*Fam76b* U937 cells. **E** The electrophoresis result of the qPCR product of the RIP experiment is in Fig. 6**D**. **F** HEK293 cells were infected by *Fam76b*-Flag-expressing lentiviruses (LV-*Fam76b*-flag). The qPCR analysis of RIP assay results for FAM76B binding to PIK3CD mRNA using LV-*Fam76b*-Flag HEK293 cells. **G** The electrophoresis result of the qPCR product of the RIP experiment in Fig. 6**F**. **H** QPCR was used to detect the efficiency of 6 pairs of PIK3CD sgRNA targeting PIK3CD mRNA. **I** Western blot of FAM76B in input and streptavidin immunoprecipitation samples of control (NC) and three well-targeted PIK3CD gRNA sets (PIK3CD sgRNA4, PIK3CD sgRNA5 and PIK3CD sgRNA6). **J** Wild-type, *Fam76b* knockout, and *Fam76b* knockout U937 cells supplemented with FAM76B after stimulation with PMA for 48 h were incubated with actinomycin D (2 μg/mL) for 0, 3, and 6 h. qPCR was used to analyze the total RNA. **P < 0.01, *Fam76b* knockout U937 vs. wild-type U937 cells; ^##^*P* < 0.01, *Fam76b* knockout + FAM76B U937 vs. *Fam76b* knockout U937 cells. The data are presented as means ± SD, *n* = 3, *P < 0.05, **P < 0.01, ***P < 0.001. *n.s*., not significant

The findings above provide evidence that FAM76B inhibits M1 macrophage polarization by regulating the PI3K/Akt/NF-κB pathway via adjusting the mRNA level of PIK3CD. To explore the potential mechanism by which FAM76B affects the PIK3CD mRNA level, an online prediction tool iDRBP_MMC (http://bliulab.net/iDRBP_MMC/result/123.139.56.76_1678448031.29) [33] was used. The prediction results showed that FAM76B may be an RNA-binding protein (Fig. 6A). Another online prediction website, catRAPID (http://big.crg.cat/gene_function_and_evolution/services/catrapid) [34], showed that FAM76B might be an RNA-binding protein, and its protein sequence contained an RNA binding domain (Fig. 6B). Moreover, catRAPID predicted that FAM76B could bind to PIK3CD mRNA (Table S4).

To further verify the predicted results, RNA immunoprecipitation sequencing (RIP-seq) was carried out in FAM76B-overexpressing U937 cells. Next, we performed a Venn analysis of the mRNAs that bind with FAM76B (screened from RIP-seq) (Table S5) and the mRNAs that are differentially expressed (as determined by RNA-seq) (Table S6). The analysis results show that PIK3CD is one of 47 genes in the combined set of sequencing results (Fig. 6C and Table S7). Then, we further verified the RIP-seq results through RIP experiments, and the RIP experimental results showed that FAM76B could bind to PIK3CD mRNA (Fig. 6D–G). We further demonstrated the interaction between FAM76B and PIK3CD mRNA using the BASU-dCasRx system (Fig. 6H, I). Considering the important role of RNA binding proteins in RNA stability, we assessed the influence of removing FAM76B on the stability of PIK3CD mRNA. *Fam76b* knockout significantly decreased PIK3CD mRNA stability, while the lost stability of PIK3CD mRNA could be rescued by overexpressing FAM76B in *Fam76b* knockout cells (Fig. 6J). The above results showed that FAM76B can bind to PIK3CD mRNA and that knocking out FAM76B can reduce the stability of PIK3CD mRNA, leading to a decrease in its level.

### FAM76B protects against IBD by regulating M1 macrophage polarization through the PI3K/Akt/NF-κB pathway

**Fig. 7:**
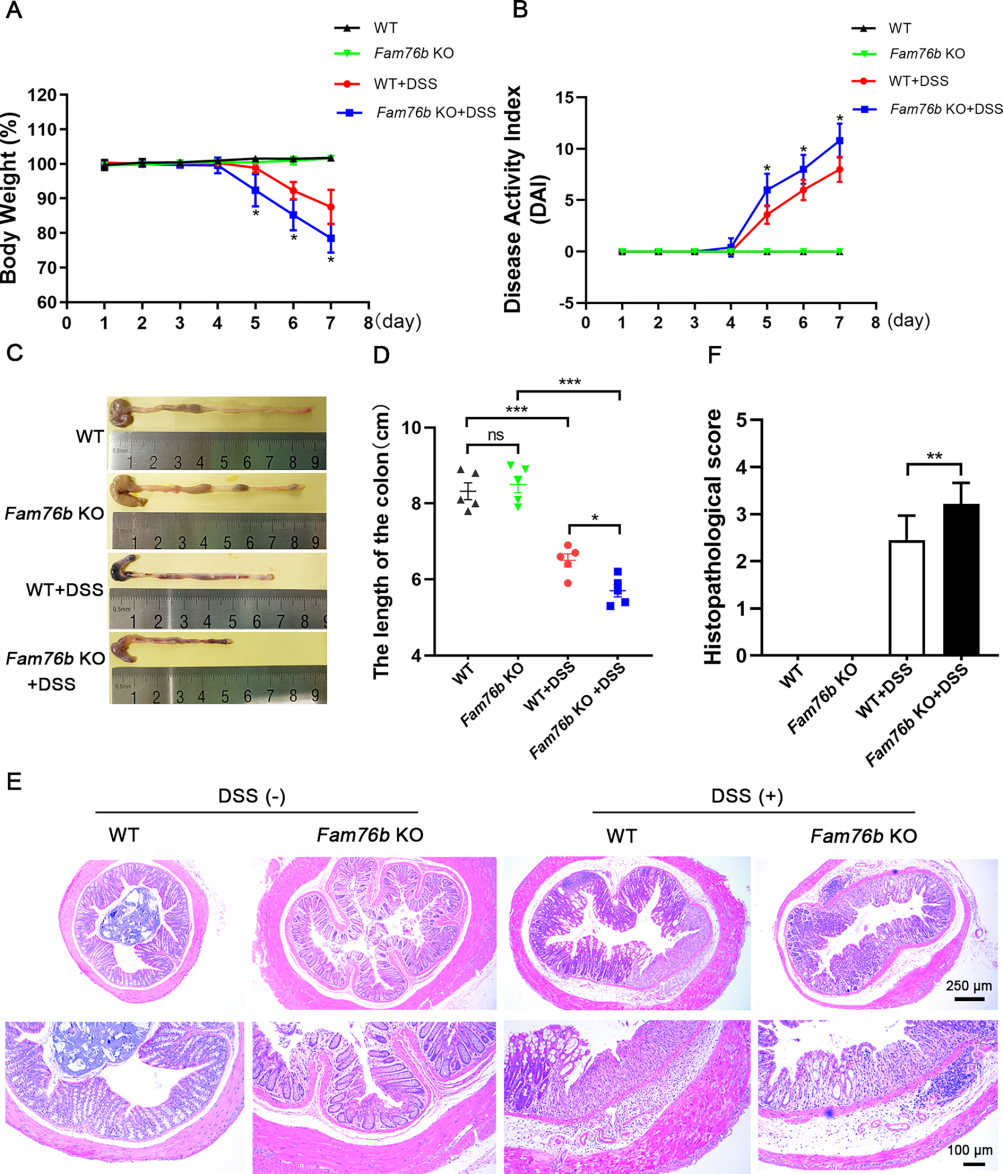
*Fam76b* knockout exacerbated DSS-induced colitis. **A** Weigh loss curves of wild-type (*n* = 5) and *Fam76b* knockout mice (*n* = 5) with/without DSS-induced colitis. **B** The DAI of wild-type and *Fam76b* knockout mice with/without DSS-induced colitis was assessed based on the consistency of fecal, fecal bleeding, and degree of weight loss, *n* = 5. **C** Representative images of the colon from wild-type and *Fam76b* knockout mice with/without DSS-induced colitis. **D** The length of the colon in wild-type and *Fam76b* knockout mice with/without DSS-induced colitis. **E** Representative histopathological images of the colon from wild-type and *Fam76b* knockout mice with/without DSS-induced colitis. Scale bar, 250 μm or 100 μm. **F** A histopathological score of histopathological images of the colon from wild-type and *Fam76b* knockout mice with/without DSS-induced colitis, *n* = 3. *P < 0.05, **P < 0.01  and ***P < 0.001. *n.s*., not significant

The in vitro experiments showed that FAM76B could inhibit the M1 polarization of macrophages via the PI3K/Akt/NF-κB pathway. Previous studies have demonstrated that IBD is intimately connected to the abnormal polarization of macrophages[21, 22]. Therefore, we next explored the influence of FAM76B on DSS-induced experimental colitis in mice in vivo. The wild-type mice and *Fam76b* knockout mice did not show significant differences in body weight, disease activity index (DAI), and colon length (Fig. 7A–D). In contrast with wild-type mice with colitis, *Fam76b* knockout mice with colitis showed more severe weight loss (Fig. 7A), higher DAI scores (Fig. 7B), and more severe shortening of the colon (Fig. 7C, D). The H&E staining of mouse colons showed normal morphology in both the wild-type and *Fam76b* knockout mice. However, the colon of *Fam76b* knockout mice with colitis showed significantly more inflammatory cell infiltration and histological damage (Fig. 7E) compared with wild-type mice with colitis. The histopathological score of the colonic tissue from wild-type and *Fam76b* knockout mice with colitis in Fig. 7E is shown in Fig. 7F. These results revealed that the knockout of *Fam76b* exacerbated the extent of colitis induced by DSS in mice.

**Fig. 8:**
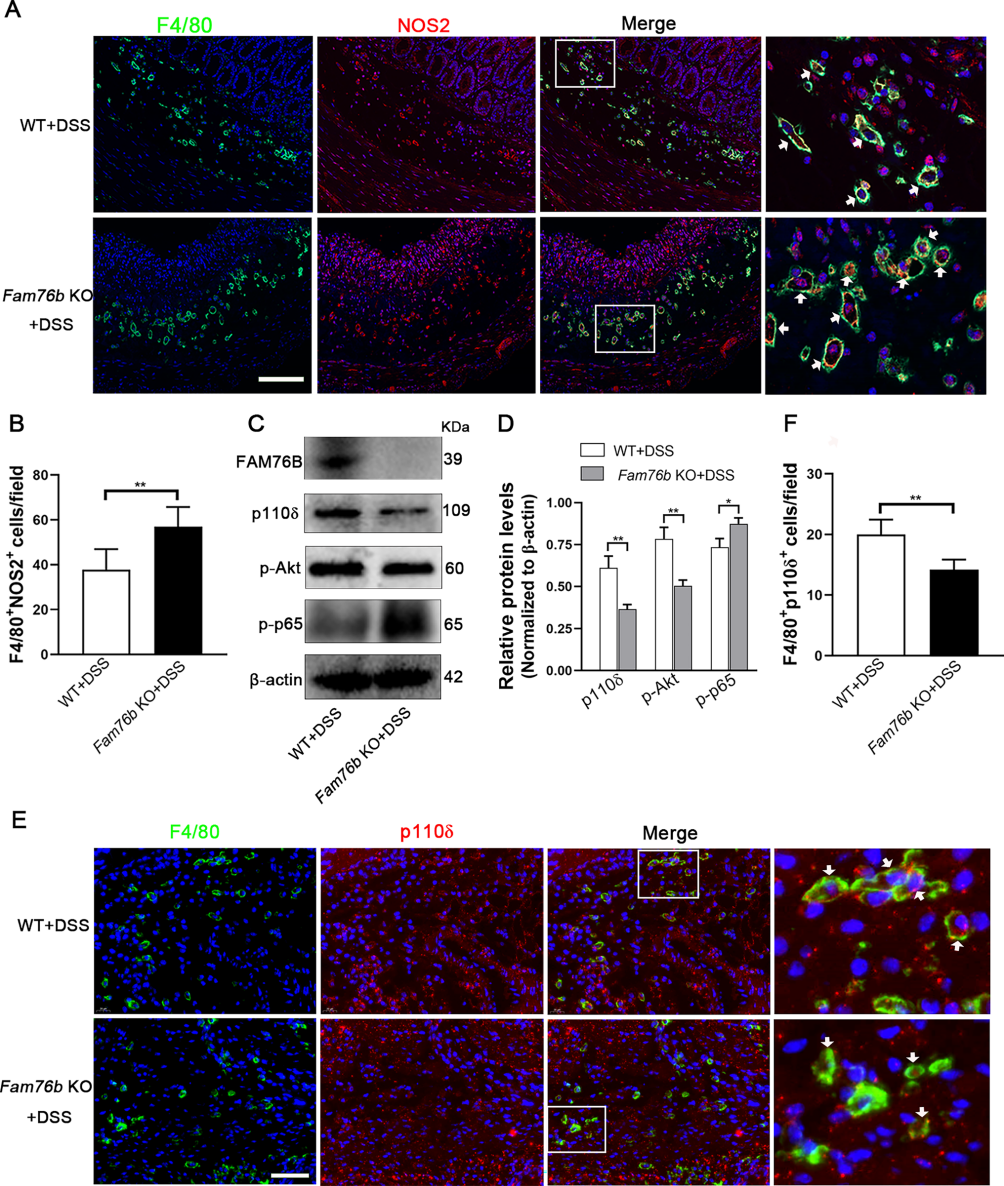
FAM76B inhibits M1 macrophage polarization by regulating the PI3K/Akt/NF-κB pathway in vivo. **A** Immunofluorescence images of F4/80 (displayed in green) and NOS2 (displayed in red) within the colonic tissues from wild-type and *Fam76b* knockout mice with DSS-induced colitis; the cell nucleus was stained using DAPI (displayed in blue). The cells indicated by the white arrows are F4/80^+^ NOS2^+^ macrophages. Scale bar, 50 μm. **B** The quantification result of the number of F4/80^+^ NOS2^+^ cells in Fig. 8**A**, n = 5. **C** The expression of FAM76B, p110δ, p-Akt, p-p65, and β-actin was measured through Western blot in the colon tissue of wild-type and *Fam76b* knockout mice with DSS-induced colitis. **D** The quantification of the p110δ, p-Akt, p-p65, and β-actin protein bands in Fig. 8**C**, n = 3. **E** Immunofluorescence images of F4/80 (displayed in green) and p110δ (displayed in red) within the colonic tissues from wild-type and *Fam76b* knockout mice with DSS-induced colitis; the cell nucleus was stained using DAPI (displayed in blue). The cells indicated by the white arrows are F4/80^+^ p110δ ^+^ macrophages. Scale bar, 50 μm. **F** The quantification result of the number of F4/80^+^ p110δ ^+^ cells in Fig. 8**E**, n = 5. *P < 0.05 and **P < 0.01

To further investigate whether the severity of colitis in *Fam7*6b knockout mice was related to the upregulated M1 macrophage polarization caused by FAM76B, we detected the expression of NOS2 in the colonic tissues of wild-type and *Fam76b* knockout mice with colitis. The immunohistochemistry results revealed that NOS2 expression in the colon tissue of *Fam76b* knockout mice with colitis was higher than that in the colon tissue of wild-type mice with colitis (Fig. S4A, quantification in Fig. S4B). A Western blot analysis confirmed this result (Fig. S4C; quantification in Fig. S4D). Then, immunofluorescent staining of F4/80 and NOS2 was employed to assess the level of NOS2 in mouse colonic macrophages. The results demonstrated that the quantity of F4/80^+^NOS2^+^M1 macrophages in the colon tissue of *Fam76b* knockout mice with colitis was significantly increased in contrast with wild-type mice with colitis (Fig. 8A, B).

Subsequently, we assessed the influence of FAM76B on the PI3K/Akt/NF-κB pathway in vivo. Western blot results demonstrated that the expression of FAM76B was deficient in *Fam76b* knockout mice (Fig. 8C). The expression of p110δ and p-Akt in the colon tissue of *Fam76b* knockout mice with colitis was decreased. P-NF-κB p65 was increased compared to wild-type mice with colitis (Fig. 8C). The quantification of the protein bands of p110δ, p-Akt, and p-NF-κB p65 is shown in Fig. 8D. Furthermore, the level of p110δ, the most critical initiation protein in the PI3K/Akt/NF-κB pathway, was measured in colon macrophages using immunofluorescent double-staining of p110δ and NOS2. The results revealed that the quantity of F4/80^+^ p110δ^+^ macrophages in the colonic tissue of *Fam76b* knockout mice with colitis was significantly lower than that in wild-type mice with colitis (Fig. 8E). Quantitation of the number of F4/80^+^ p110δ^+^ cells in Fig. 8E was shown in Fig. 8F. The in vivo findings above demonstrated that FAM76B can exert a protective effect against colitis by regulating M1 macrophage polarization via the PI3K/Akt/NF-κB pathway.

## Discussion

**Fig. 9:**
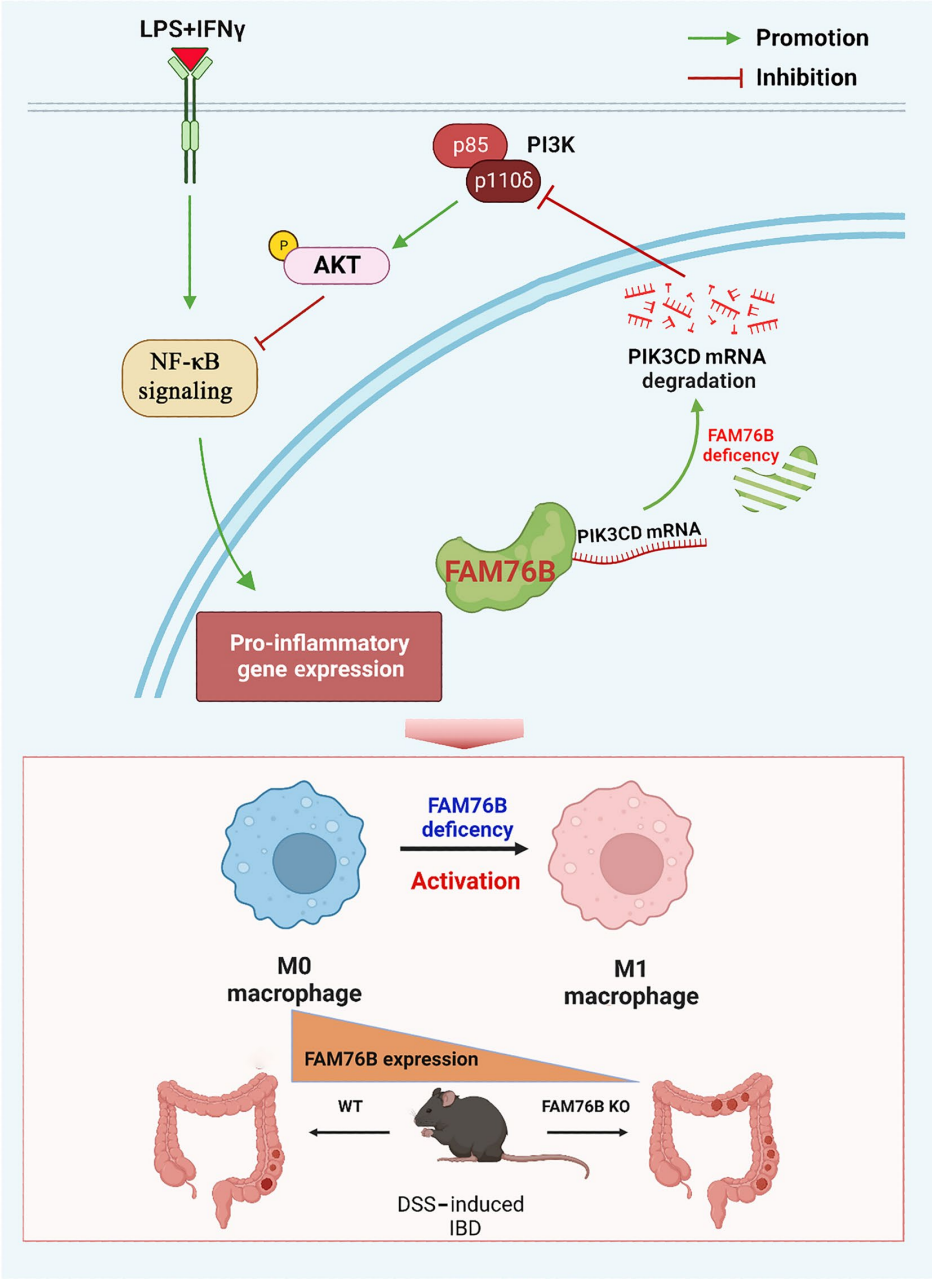
FAM76B is essential for inhibiting M1 macrophage polarization and protecting against IBD. Under normal conditions, FAM76B binds to PIK3CD mRNA. When FAM76B is knocked out, PIK3CD mRNA becomes less stable, which results in a reduction in the expression of p110δ (the catalytic subunit of PI3K). Decreasing p110δ expression results in a decrease in downstream p-Akt protein levels and an increase in protein levels of its crosstalk partner, p-NF-κB p65, causing macrophages to polarize from the M0 to M1 phenotype. Moreover, the expression of FAM76B in M1 macrophages is significantly downregulated. In vivo, FAM76B protects against IBD by inhibiting M1 macrophage polarization through the PI3K/Akt/NF-κB pathway

In this study, we have demonstrated for the first time that FAM76B can regulate PI3K/Akt/NF-κB-mediated M1 macrophage polarization by stabilizing PIK3CD mRNA (Fig. 9). We first found that more M1 macrophages were present in *Fam76b* knockout U937 cells vs. wild-type U937 cells, that FAM76B expression in M1 macrophages was significantly decreased, and that M1 macrophage-associated marker expression increased after *Fam76b* knockout. These findings suggested that FAM76B plays a vital role in regulating M1 macrophage polarization. Moreover, the function of FAM76B in inhibiting M1 macrophage polarization to protect against IBD was further demonstrated in a *Fam76b* knockout experimental colitis mouse model. These findings potentially indicate a significant contribution of FAM76B in inflammation-related diseases.

Studies have reported that BMMs from p110δ-deficient mice exhibit an increased inflammatory response to TLR signaling, manifested by higher levels of inflammatory cytokines and nitric oxide, which led to mild colitis in p110δ-deficient mice at 8 weeks of age [35, 36]. In the KEGG enrichment analysis examining wild-type vs. *Fam76b* knockout U937 cells with the M1 phenotype, we observed that the PI3K/Akt pathway was highly enriched in M1 *Fam76b* knockout U937 cells. Moreover, the level of PIK3CD mRNA significantly decreased after *Fam76b* knockout. PIK3CD is the encoding gene of the catalytic subunit p110δ of PI3K. Studies have demonstrated that PIK3CD overexpression increases the level of p-Akt, while PIK3CD inhibition shows the opposite result [18]. In this study, we also found that *Fam76b* knockout downregulated PIK3CD mRNA levels, resulting in a decrease in the protein level of p110δ and its downstream p-Akt, as well as an increase in the protein level of p-NF-κB p65. This finding accords with those of previous studies investigating the impact of PI3K/Akt on NF-κB expression [37–39]. However, after FAM76B supplementation, this change was rescued. Moreover, the supplementation of FAM76B caused a decrease in NOS2 expression: the M1 macrophage polarization caused by *Fam76b* knockout was reversed after supplementing *Fam76b* knocked out U937 cells with FAM76B. These findings suggest that FAM76B regulates M1 macrophage polarization through the PI3K/Akt/NF-κB signaling axis.

In this study, FAM76B was confirmed to affect the level of PIK3CD mRNA. We did consider that FAM76B may affect the transcription of PIK3CD as a DNA-binding protein or by interacting with DNA-binding proteins, maybe as an RNA-binding protein or by interacting with RNA-binding proteins, affecting the post-transcriptional regulation of PIK3CD mRNA. Bioinformatics predictions were performed to test this idea, which indicated that FAM76B may be an RNA-binding protein and could bind to PIK3CD mRNA. We verified the predicted results by RIP and the BASU-dCasRx system, and the results showed that FAM76B could indeed bind to PIK3CD mRNA. Studies have found that RNA-binding proteins can affect the biological function of post-transcriptional RNA by regulating the stability, alternative splicing, polyadenylation, and translation of mRNA, thereby regulating and controlling the expression of genes [40]. Ensuring RNA stability is an important way for RNA-binding proteins to modulate gene expression. In the process of exploring the downregulation of the PIK3CD mRNA by *Fam76b* knockout, we found that knocking out FAM76B could decrease the stability of PIK3CD mRNA, while the stability of PIK3CD mRNA increased after FAM76B supplementation.

The development and outcome of IBD have a close association with the phenotypic transformation of colonic macrophages [41–43]. Considering the intimate connection between macrophage polarization and IBD, a DSS-induced IBD model was used in this study. Under physiological conditions, no significant differences were observed in body weight, colon length, and colonic histological morphology between wild-type and *Fam76b* knockout mice, possibly related to the fact that, under physiological conditions, signaling pathways involved in the disease state are in a non-activated state. Additionally, in Fig. 7E, we observed that the colon of the DSS (–) WT group seems smaller compared to the other three groups. This discrepancy may be attributed to innate differences in the colon of mice. In the DSS-induced colitis model, we found that *Fam76b* knockout mice with DSS-induced colitis had more pronounced symptoms of colitis than wild-type mice with DSS-induced colitis, indicating that the loss of FAM76B exacerbates the severity of experimental colitis in mice. Moreover, more inflammatory cell infiltration and more severe epithelial damage were observed in the colon tissues of *Fam76b* knockout mice with colitis in comparison to wild-type mice with colitis. These changes were related to the increase in M1 macrophages and the change of the PI3K/Akt/NF-κB pathway after *Fam76b* knockout. We concluded that FAM76B inhibited M1 macrophage polarization by regulating the PI3K/Akt/NF-κB signaling axis and protected against IBD.

In conclusion, our study showed that FAM76B could bind to PIK3CD mRNA. *Fam76b* knockout reduced the stability of PIK3CD mRNA, then regulated the PI3K/Akt/NF-κB pathway, promoted M1 macrophage polarization, and led to *Fam76b* knockout mice being susceptible to DSS-induced experimental colitis. These findings offer novel perspectives into the mechanism underlying macrophage polarization and suggest that FAM76B may serve as a prospective treatment target for IBD.

### Supplementary Information

Below is the link to the electronic supplementary material.

Supplementary file1 (DOCX 1144 KB)

Supplementary file2 (XLSX 4519 KB)

Supplementary file3 (XLS 807 KB)

Supplementary file4 (XLS 213 KB)

Supplementary file5 (XLS 21 KB)

## Data Availability

Supplementary data to this article can be found online.
