# Supplementary material for: FAM76B regulates PI3K/Akt/NF-κB-mediated M1 macrophage polarization by influencing the stability of PIK3CD mRNA
Source: Cell Mol Life Sci. 2024 Feb 29;81(1):107. doi: 10.1007/s00018-024-05133-2 (PMC10904503; doi:10.1007/s00018-024-05133-2)
Supplement: Supplementary file 1 — Supplementary file1 (DOCX 1144 KB) [file 18_2024_5133_MOESM1_ESM.docx]

FAM76B regulates PI3K/Akt/NF-κB -mediated M1 macrophage polarization by influencing the stability of PIK3CD mRNA

Juan Wang^1^, Xinyue Zhao^1^, Qizhi Wang^1^, Xiaojing Zheng^1^, Dilihumaer Simayi^1^, Junli Zhao^1^, Peiyan Yang^1^, Qinwen Mao^2^, Haibin Xia^1*^

*^1^ Laboratory of Gene Therapy, Department of Biochemistry, College of Life Sciences, Shaanxi Normal University, Xi’an 710062, P.R. China*

*^2^ Department of Pathology, University of Utah, Huntsman Cancer Institute, 2000 Circle of Hope Drive, Salt Lake City, UT 84112, USA*

*Corresponding author:

Haibin Xia, Ph.D., M.D.

Laboratory of Gene Therapy

Department of Biochemistry

College of Life Sciences

Shaanxi Normal University

199 South Chang’an Road

Xi’an 710062

Shaanxi Province, P.R. China

E-mail: [hbxia2001@163.com](mailto:hbxia2001@163.com); [xiahaibin@snnu.edu.cn](mailto:xiahaibin@snnu.edu.cn)

**Table S1.** The sequences of the primers used for *q*PCR in the study.

| Primer | Sequence (Forward) | Sequence (Reverse) |
| --- | --- | --- |
| human-*Fam76b* | AGCAGATAGTGGGGGAACAGACAA | TTTTGGCCTGAAGTTGTTCCACAGT |
| human-*IL6* | GGATTCAATGAGGAGACTTGCC | TGGCATTTGTGGTTGGGTCA |
| human-*IL1β* | TGGCAATGAGGATGACTTGT | GTGGTGGTCGGAGATTCGTA |
| human-*TNFα* | TCCCCAGGGACCTCTCTCTA | GAGGGTTTGCTACAACATGGG |
| human-*IL23A* | CTCAGGGACAACAGTCAGTTC | ACAGGGCTATCAGGGAGCA |
| human-*PIK3CD* | AACCTCAGCACCATCAAGCA | TCAAACTCGTGGAGGCCTTT |
| human-*GAPDH* | GCACCGTCAAGGCTGAGAAC | TGGTGAAGACGCCAGTGGA |
| mouse-*Fam76b* | ACTGTGGAACAACTCCAGGCCAA | CAGGGAGATGTTAGCACGCTTCCA |
| mouse-*NOS2* | CAACATCAGGTCGGCCATCACT | ACCAGAGGCAGCACATCAAAGC |
| mouse-*IL6* | CCTTCCTACCCCAATTTCCAAT | GCCACTCCTTCTGTGACTCCAG |
| mouse-*IL1β* | AATCTCGCAGCACATCA | AAGGTGCTCATGTCCTCATC |
| mouse-*GAPDH* | AAGGCCGGGGCCCACTTGAA | AGCAGTTGGTGGTGCAGGATGC |

**Table S2.** The sequences of the primers used for *PIK3CD* sgRNA in the study.

| Primer | Sequence (Forward) | Sequence (Reverse) |
| --- | --- | --- |
| h*PIK3CD* sgRNA1 | AAACGAAGTCAACCACAACGCTCTGAT | AAAAATCAGAGCGTTGTGGTTGACTTC |
| h*PIK3CD* sgRNA2 | AAACCCCTCAAACTTAACGTTGACCAG | AAAACTGGTCAACGTTAAGTTTGAGGG |
| h*PIK3CD* sgRNA3 | AAACGCAGATGTTGATGTCGAACTCCA | AAAATGGAGTTCGACATCAACATCTGC |
| h*PIK3CD* sgRNA4 | AAACCTTCTCGTGCTCATACAGCTCCC | AAAAGGGAGCTGTATGAGCACGAGAAG |
| h*PIK3CD* sgRNA5 | AAACCCGTTCTTAAAGATGATGCCCAC | AAAAGTGGGCATCATCTTTAAGAACGG |
| h*PIK3CD* sgRNA6 | AAACGCCAAAATCAATGTGGAACAGCT | AAAAAGCTGTTCCACATTGATTTTGGC |

**Table S3.** The sequences of the primers for mouse genotyping in the study.

| Primer | Sequence |  |
| --- | --- | --- |
| Mouse *Fam76b* genotyping | Forward | GCAGAGATTGGGTGCAGACT |
|  | Reverse | GAGACCCAATCTCACTCTTTG |
|  | V76 Reverse | CCAATAAACCCTCTTGCAGTTGC |

**Figure S1**

**
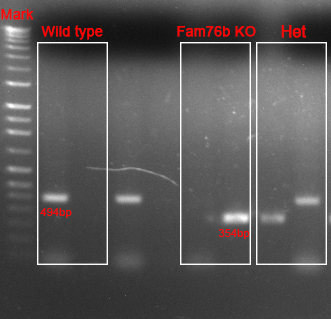
**

**Figure S1.** Electrophoretic image of PCR products for genotype identification of mice. (Het: Heterozygous).

**Figure S2**


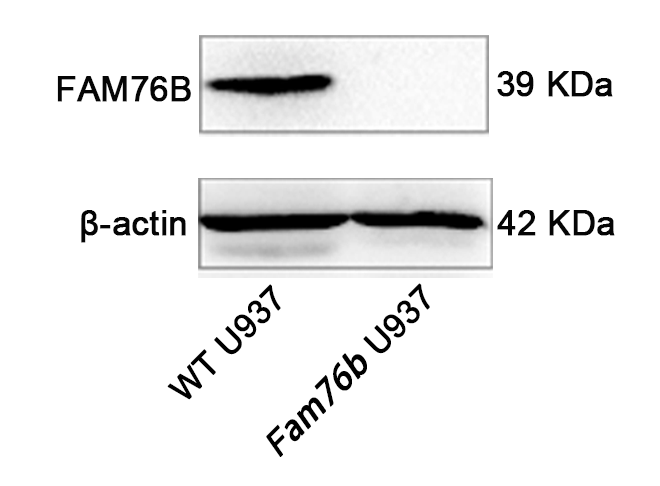


**Figure S2.** The protein level of FAM76B in wild-type and *Fam76b* knockout U937 cells was detected by Western blot.

**Figure S3**


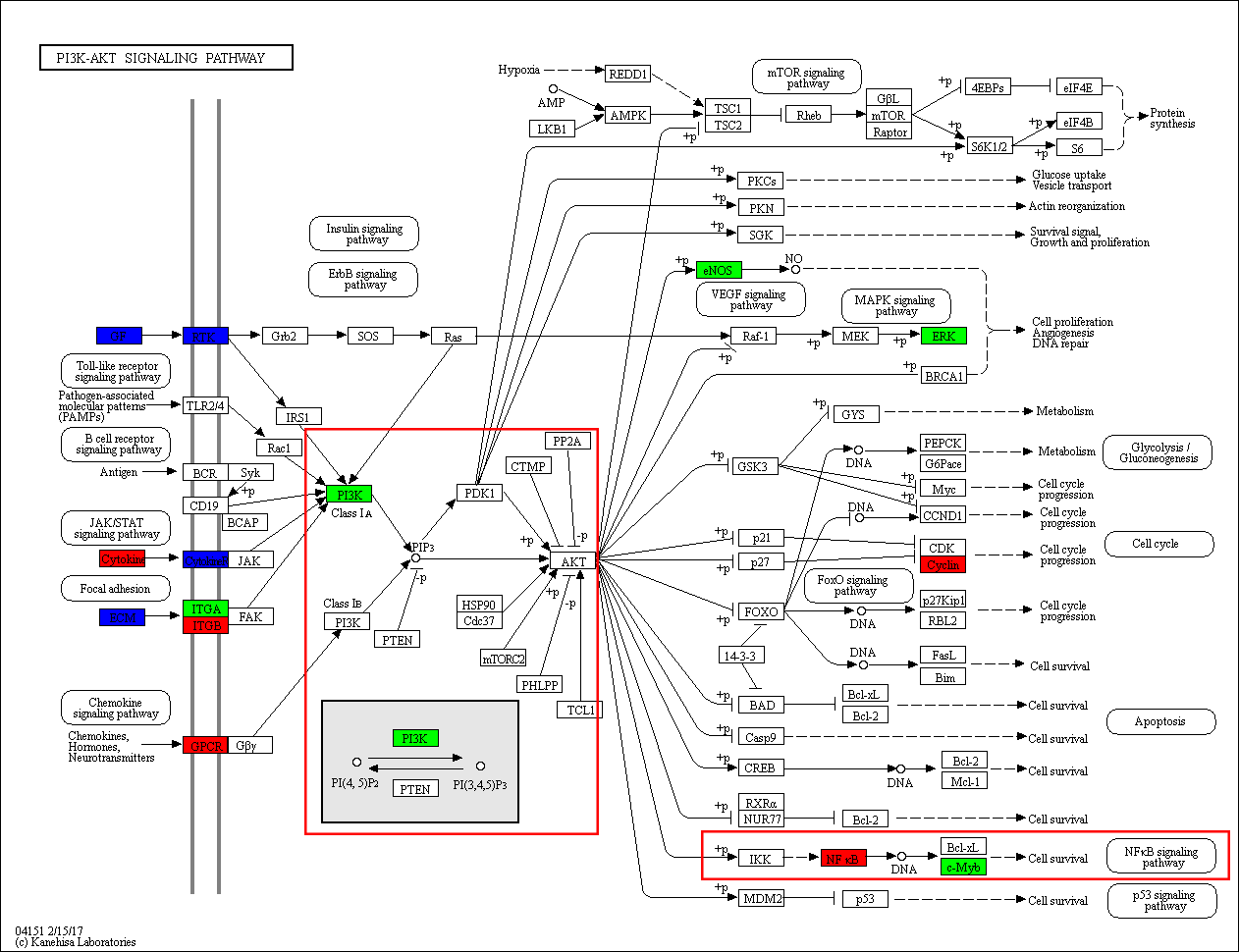
**Figure S3.** PI3K/Akt signaling pathway map showed that the mRNA level of NF-κB with crosstalk with the PI3K/Akt signaling pathway increased significantly after *Fam76b* knockout (Red shows an increase, green shows a decrease, Blue shows an increase or a decrease, Genes with no colors shows an unchanged).

**Figure S4**


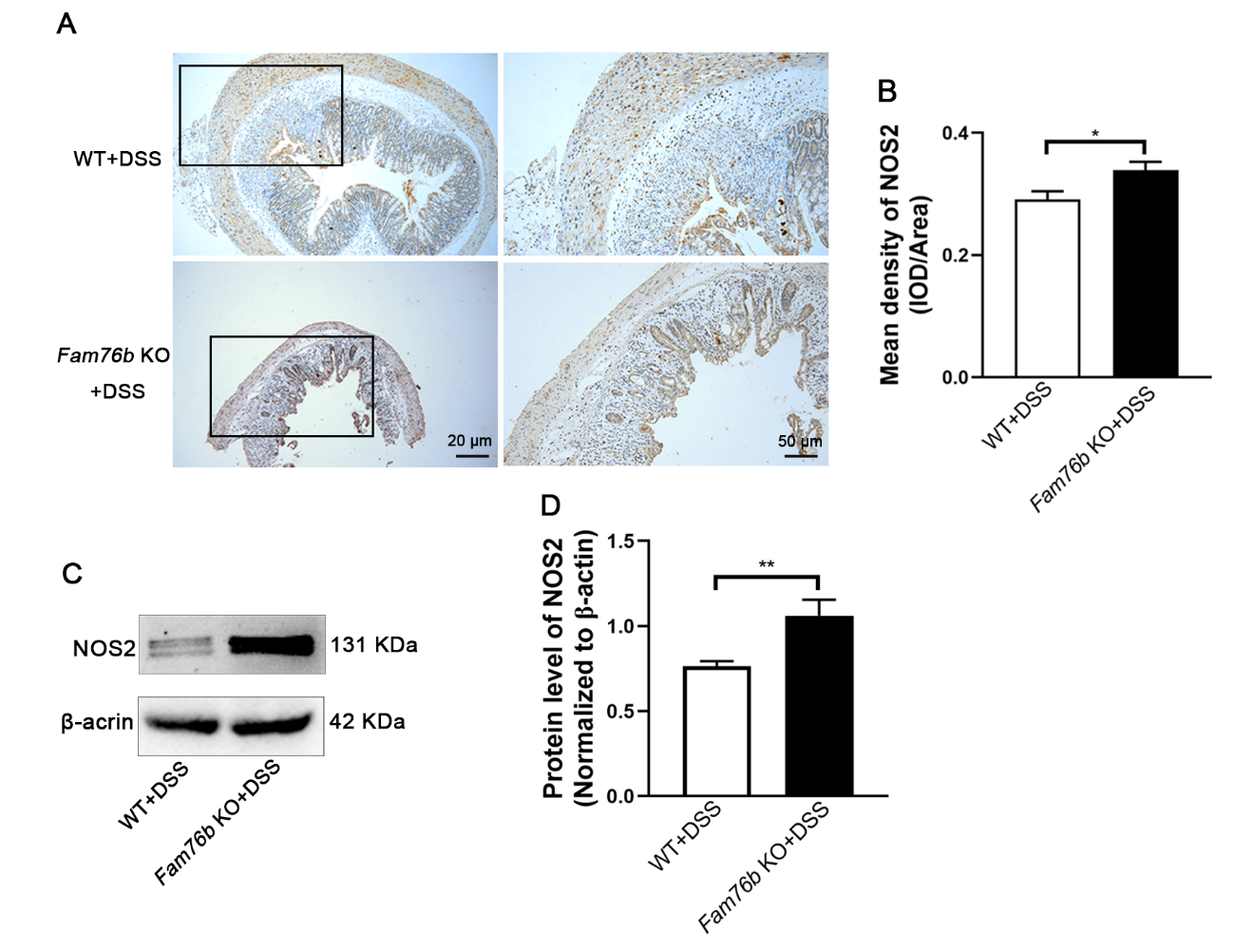


**Figure S4.** FAM76B inhibits M1 macrophage polarization *in vivo*. (**A**) Representative immunohistochemical images of the colon from wild-type and Fam76b knockout mice with DSS-induced colitis. (**B**) The quantification result of the NOS2 immunohistochemical staining in Figure S4A, IOD stand for Integrated Option Density. (**C**) The expression of NOS2 was measured using Western blot in the colon tissue of wild-type and Fam76b knockout mice with DSS-induced colitis. (**D**) The quantification result of the NOS2 protein bands in Figure S4C. The data are presented as means ± SD, *n* = 3, **P* < 0.05 and ***P* < 0.01.
